# Two birds with one stone: geriatric competency learning promotes hidden curriculum in the era of COVID-19

**DOI:** 10.1080/10872981.2022.2035201

**Published:** 2022-02-03

**Authors:** Jyotsna Pandey, Brenda Varriano, Andrea Beatty

**Affiliations:** a Foundations of Medicine,Central Michigan University (CMU) College of Medicine, Mt. Pleasant, Michigan; b Healthy Aging, Central Michigan University (CMU) College of Medicine, Mt. Pleasant, Michigan, USA

**Keywords:** Hidden curriculum, geriatric competency, self-care capacity, rural community health, service learning, leadership skills, medical education

COVID-19 pandemic required community shutdowns that had profound implications on medical education when pre-clerkship instruction shifted to a web-based format [1,2]. Virtual learning lacks personal contact and abrogates observational learning that comes from ‘the hidden curriculum (HC)’[3]. A great deal of what is learned in medical school takes place not within formal course offerings but within medicine’s HC [4]. HC is defined as a set of influences that function at the organizational cultural level to impact learning. Although not formally stated, it is conveyed through role modeling, social interactions, and unintentional observations, i.e., of the words said or omitted, jokes told, silences observed by the learner [5,6]. HC can enhance learning components like leadership, communication, and management skills; collaboration with the wider community; capacity and cultures; and student activities [7]. Students recognize that many components required during the clinical years that enhance the breadth and depth of medical education are acquired through the HC [6]. Unlike the explicit declared curriculum, HC is implicit and includes the daily socialization process, where norms and values are transmitted during delivery of the curriculum [3,8,9]. This happens due to intentional and unintentional social interactions among the learners and other stakeholders of the learning community and influence learner’s development [7]. Many of these social interactions were blunted when the COVID-19 pandemic necessitated the curriculum delivery approach move to virtual platforms [1,2,10], redefining, and somewhat nullifying the HC learning.

The expanding geriatric population in the US requires an emphasis on geriatrics training for all health professionals [11]. Older adults’ wellbeing was further compromised during COVID-19 pandemic due to a poorer prognosis if infected further emphasizing the importance of geriatric healthcare and geriatric competency training. Competency-based education has been proposed to prepare medical students to address the needs of this vulnerable population with emphasis on healthcare and prevention [11]. Rural older adults (ROA) have unique wellbeing and health issues compared to their urban counterparts and are often financially constrained [12]. COVID-19 pandemic also brought out the unique hardships faced by the rural dispersed populations, not just due to the forced isolation but due to the suspension of community-based social projects and social contact which also impacted medical education [10].

We envisioned that a virtual service-learning experience and a virtual community-based novel learning intervention could address both issues.

The initiative CMU-CARES (Central Michigan University-Crisis Avoidance for Rural Elderly stakeholders) is a summer research project that involved medical students and rural older adults collaborating to develop a ‘crisis preparedness toolkit’ with an aim to disseminate it to the community. It provides an important component of learning a geriatric competency, i.e., self-care capacity.

The project leadership team of five second-year medical students supervised by a faculty met virtually once a week. Twenty ROA participants were recruited by advertising for volunteers through senior centers in rural, resource-poor, central Michigan counties. A semi-structured interview format modeled on the World Health Organization Quality of Life-BREF [13] was developed to elicit their experiential knowledge and hardships faced during the COVID-19 pandemic. Virtual interviews were conducted using the telephone and recorded with older adults’ consent. Recordings were transcribed using a WebEx application. Each transcribed script was cross-checked with audio by two members of the team for accuracy. Qualitative data analysis was done by NVivo software. Open coding scheme was used to identify frequently occurring concepts to create codes. Each script was coded independently by three student team members. On compilation, similar concepts were grouped, and eight themes emerged. A density analysis was done to determine the relative importance of each theme.

Preliminary themes identified include (1) *Isolation and Interpersonal relationships; (2) Concerns for others; (3) Lifestyle requirements; (4) Medical requirements; (5) Technology literacy; (6) Community involvement; (7) Wellbeing; and (8) Information gathering*. These core themes will guide the development of ‘crisis preparedness toolkit’ that fosters self-care capacity, resilience, and empowers self-reliance in a crisis. The implementation phase will involve the dissemination of the toolkit to the community and development of materials to educate the community of its value in a crisis. The educational pamphlets and presentations for dissemination will be developed by teams of 4–5 students involving the full second-year medical student body. All processes of collective decision-making during the design, development and implementation of the project have student involvement and promote competency in methods that improve geriatric self-care capacity. Direct interaction with older adults provides an important understanding of the older adults’ perspective on self-care and wellbeing needs that enhance the core geriatric competency training [11]. CMU-CARES, thus, supports the geriatric curriculum, and the community-based project acts as a prototype for future physicians to understand and address the needs of vulnerable and underserved communities [14,15].

Through interactions with ROA, medical students can appreciate the vulnerability of this population, and challenges and barriers they face in maintaining wellbeing. This valuable learning experience may shape students’ future interactions with older adults. Although not a course requirement, students learn through role-modeling, gain leadership skills, and develop communication skills to interact and appreciate the issues at hand. This combination of learning interactions with the geriatric population not only addresses a curricular gap but also supports learning through the HC [7,16]. CMU-CARES also provided digital proficiency through problem-solving to perform virtual interviews, when in-person interactions were limited. The use of technology to interact with ROA provided medical students with virtual interview experience, an increasingly valuable skill as telemedicine gains relevance [17–19].

**Next Steps**: The pandemic caused a challenge by abrogating skills learned through HC; a student-led service-learning project CMU-CARES provided a solution. We propose that student-led service-learning projects can be used, even in a virtual teaching environment, to enhance competencies normally learned through the HC and dependent on social contact. This service-learning project not only empowers ROA to help gain resilience, and self-sufficiency in a crisis, it also prepares students for a role in community health education to improve this population’s self-care capacity. It also enhances their appreciation for the circumstances, challenges, and barriers experienced by older adults. It provides HC learning (leadership and management; collaboration with the wider community; capacity and cultures) that comes from personal interactions and socialization with peers and community. Future studies will follow this student team into clerkship and evaluate their self-assessed level of preparedness to interact with older adults as patients. Finally, based on the themes identified in the interviews, a toolkit is in development that will be used in the implementation phase involving the full class of second-year medical students.

## Declaration

A determination of exempt status was made by the Central Michigan University Institutional review board. IRB # 2020-656
